# Overrepresentation of Germline Immune-Related Gene Variants in Patients with Acquired Bone Marrow Failure

**DOI:** 10.1007/s10875-025-01951-6

**Published:** 2025-11-05

**Authors:** Zuzana Pinc, David Kundrat, Monika Kaisrlikova, Andrea Hrustincova, Sarka Vanikova, Iva Trsova, Jitka Vesela, Martin Vostry, Barbora Pejsova, Sarka Ransdorfova, Lucie Slamova, Tomas Prochazka, Daniel Lysak, Anna Jonasova, Marketa Stastna Markova, Jaroslav Cermak, Monika Belickova, Hana Votavova

**Affiliations:** 1 Institute of Hematology and Blood Transfusion, Prague, Czech Republic; 2 First Faculty of Medicine, Charles University, Prague, Czech Republic; 3 Faculty of Science, Charles University, Prague, Czech Republic; 4 Department of Hematology and Oncology, University Hospital Pilsen, Pilsen, Czech Republic; 5 First Department of Medicine, First Faculty of Medicine, Charles University and General University Hospital, Prague, Czech Republic; 6 Institute of Clinical and Experimental Hematology, First Faculty of Medicine, Charles University, Prague, Czech Republic

**Keywords:** Aplastic anemia, Hypoplastic myelodysplastic neoplasms, Mutational landscape, T cell transcriptome

## Abstract

**Purpose:**

Bone marrow failure (BMF) in idiopathic aplastic anemia (AA) and hypoplastic myelodysplastic neoplasms (MDS-h) results from the destruction of hematopoietic progenitors by autoreactive T cells; however, the molecular events driving the pathogenesis of these disorders remain unclear. We therefore applied whole-exome sequencing (WES) in AA and MDS-h patients to identify acquired and inherited gene variants presumed to have functional consequences for BMF. We also used transcriptome profiling to investigate the molecular mechanisms underlying the aberrant T cell response.

**Methods:**

WES was performed on DNA from 42 patients at diagnosis. Transcriptome profiling of CD3⁺ cells was conducted in 21 patients and 10 healthy donors. Peripheral blood cell populations were analyzed by flow cytometry.

**Results:**

Pathogenic/likely pathogenic (P/LP) somatic gene variants were detected in 79% of patients and were functionally associated with BMF-relevant processes such as antigen processing/presentation, T cell-mediated immunity, and DNA repair. P/LP germline gene variants were found in all patients, almost half of whom harbored variants associated with inborn errors of immunity. Patient T cells displayed expression signatures of increased inflammation, apoptosis, hypoxia response, and decreased oxidative phosphorylation. Dysregulated long noncoding RNAs were predicted to primarily regulate the differentiation of T helper 17 cells. Patients also showed significantly lower frequencies of immature progenitors and natural killer cells compared with controls.

**Conclusion:**

Patients with idiopathic AA and MDS-h carried multiple germline immune-related gene variants that may increase susceptibility to immune-mediated BMF. Furthermore, patient T cells exhibited altered energy metabolism, which may represent a therapeutic target for modulating immune responses in autoimmune diseases.

**Supplementary Information:**

The online version contains supplementary material available at 10.1007/s10875-025-01951-6.

## Introduction

Idiopathic aplastic anemia (AA) and hypoplastic myelodysplastic neoplasms (MDS-h) are rare bone marrow failure (BMF) disorders characterized by pancytopenia and hypocellular bone marrow (hypo-BM) [1]. Although AA and MDS-h represent distinct clinical entities, they share a common pathophysiology linked to damage to hematopoietic stem and/or progenitor cells (HSPCs) by autoreactive T cells. The T cells are activated by the recognition of as yet unidentified autoantigens, undergo clonal expansion and overproduce proinflammatory cytokines [2]. However, the molecular mechanisms underlying the complex pathogenesis of these disorders are still not fully understood [3, 4].

Next-generation sequencing (NGS) has rapidly increased our knowledge of the mutational landscape of myelodysplastic neoplasms (MDS). Up to 20% of acquired AA (aAA) patients and 35% of MDS-h patients were found to have at least one MDS-associated somatic mutation; however, no specific pattern of mutations discriminating these disorders has been defined [5, 6]. Somatic mutations in the *PIGA* and *BCOR/BCORL1* genes appear to be more specific for aAA, but they are also found in MDS-h. Clones carrying these mutations tend to remain stable or even disappear and are associated with a better response to immunosuppressive therapy (IST), and longer overall survival in aAA patients. In contrast, somatic mutations in the *DNMT3A* and *ASXL1* genes are associated with increasing clone size and worse clinical outcomes [7, 8].

Despite advances in treatment, many patients with acquired BMF syndromes (aBMFS) experience adverse outcomes due to the intricate biology of these disorders. To improve this, we applied whole-exome sequencing (WES) in AA and MDS-h patients to identify pathogenic somatic and germline gene variants, focusing on non-MDS-associated variants that are presumed to have functional consequences for immune-mediated BMF. Using transcriptome profiling of patient T cells, we further studied the molecular mechanisms underlying the abnormal T cell response.

## Methods

### Study Cohort

The study cohort consisted of 55 patients with idiopathic AA (*n* = 37) or MDS-h (*n* = 18) at diagnosis, and 10 healthy blood donors. The patient characteristics are summarized in Table S1. In AA patients, the diagnosis, disease subtype and immunosuppressive therapy (IST) response were assessed according to established guidelines for the diagnosis and management of adult AA [9]. The AA patients had nonsevere AA (NSAA, *n* = 12), severe AA (SAA, *n* = 18), or very severe AA (VSAA, *n* = 7). A total of 24 patients were treated with IST, and their response was evaluated after a 6-month follow-up (Table S2). Eighteen of the 24 evaluable patients responded after 6 months of treatment (ORR = 75%), including 12 complete responses (CRs) (50%). The distribution of AA subtypes did not differ significantly between IST responders and non-responders. All patients with MDS included in the study fulfilled the diagnostic criteria for MDS-h according to the 2022 World Health Organization classification of myeloid neoplasms [10]. The IPSS-R scores of the MDS-h patients were very low (*n* = 3), low (*n* = 9), or intermediate (*n* = 6) [11]. This study followed the ethical standards of the World Medical Association’s Declaration of Helsinki, and the protocol was approved by the Institutional Scientific Board and the Ethics Committee of the Institute of Hematology and Blood Transfusion. All enrolled patients and blood donors provided written informed consent.

### Cell and Nucleic Acid Isolation

CD3^+^ cells were isolated from peripheral blood (PB) by magnetic separation (Miltenyi Biotec, Bergisch Gladbach, Germany) according to the manufacturer’s instructions. Genomic DNA was isolated from bone marrow (BM) cells using MagCore Plus II (RBC Bioscience, New Taipei City, Taiwan) and from buccal swabs (BS) using ISOHELIX™ DNA/RNA Buccal Swabs-SK-2 S (Cell Projects Ltd, Harrietsham, Kent, UK) according to the respective manufacturer’s instructions. Total RNA isolation was performed using acid guanidinium thiocyanate-phenol-chloroform extraction. The concentration of nucleic acids was quantified on Qubit 3.0 fluorometer (Life Technologies, Carlsbad, CA, USA), and the quality was determined with Agilent 4200 TapeStation (Agilent Technologies, Santa Clara, CA, USA) and NanoDrop One Spectrophotometer (Thermo Fisher Scientific, Waltham, MA, USA).

### DNA Sequencing

WES was performed on genomic DNA from BM samples collected from 42 hypo-BM patients (8 NSAA, 16 SAA, 5 VSAA, and 13 MDS-h patients) at diagnosis and matched BS samples to verify the germline origin of the gene variants. Whole-exome libraries were constructed using SureSelect Human All Exon V7 kit (Agilent Technologies) and sequenced on NovaSeq 6000 analyzer (Illumina, San Diego, CA, USA). Data analysis, including alignment to the hg19 human reference genome, variant calling, filtering, and annotation, was completed using VarSome Clinical platform [12]. Only pathogenic (P) and likely pathogenic (LP) variants were analyzed further. Details of variant classification and sequencing data deposition are available in Supplementary Methods S1.

Additionally, targeted sequencing of 54 myeloid-associated genes was performed on a subset of 20 BM samples using MiSeq platform as described previously [13].

### RNA Sequencing

Transcriptome profiling was performed for PB T cells isolated from 21 hypo-BM patients (5 NSAA, 8 SAA, 2 VSAA, and 6 MDS-h patients) collected at diagnosis, prior to the initiation of treatment, and from 10 age-matched healthy controls. The median age of the patients and controls was 48 and 45 years, respectively. The library was constructed using NEBNext Ultra II Directional RNA Library Prep Kit (NEB, Ipswich, MA, USA). Sequencing was performed on NovaSeq 6000 analyzer (Illumina), and the raw data were processed and analyzed according to *Kaisrlikova* et al. [13].

### Seahorse Assay

Mitochondrial respiration was measured in CD3^+^ cells from 3 hypo-BM patients and 3 healthy controls using Seahorse XFp Analyzer and Seahorse XFp Cell Mito Stress Test Kit (Agilent Technologies). Details of the assay are described in Supplementary Methods S1.

### Flow Cytometry

The frequencies of specific PB cell subpopulations were determined in 14 hypo-BM patients (2 NSAA, 6 SAA, 1 VSAA, and 5 MDS-h patients) and 6 healthy controls using flow cytometry. Details of sample processing are described in Supplementary Methods S1 and Table S3. The samples were measured on BD FACSymphony A5 cytometer, and the data were analyzed using FlowJo 10.8.1 software (BD Biosciences, Milpitas, CA, USA). The cell populations were defined as shown in Figure S1.

### Statistical Analysis

Statistical analyses were performed with GraphPad Prism v9 (GraphPad Software Inc., San Diego, CA, USA). Student’s t-test was used to determine significant differences between groups, and a p value of less than 0.05 was considered to indicate statistical significance. Correlations were calculated using the Pearson and Spearman correlation methods (package stats) and then plotted using the ggplot2 package in R software. 

## Results

### Somatic Mutation Landscape

**Fig. 1:**
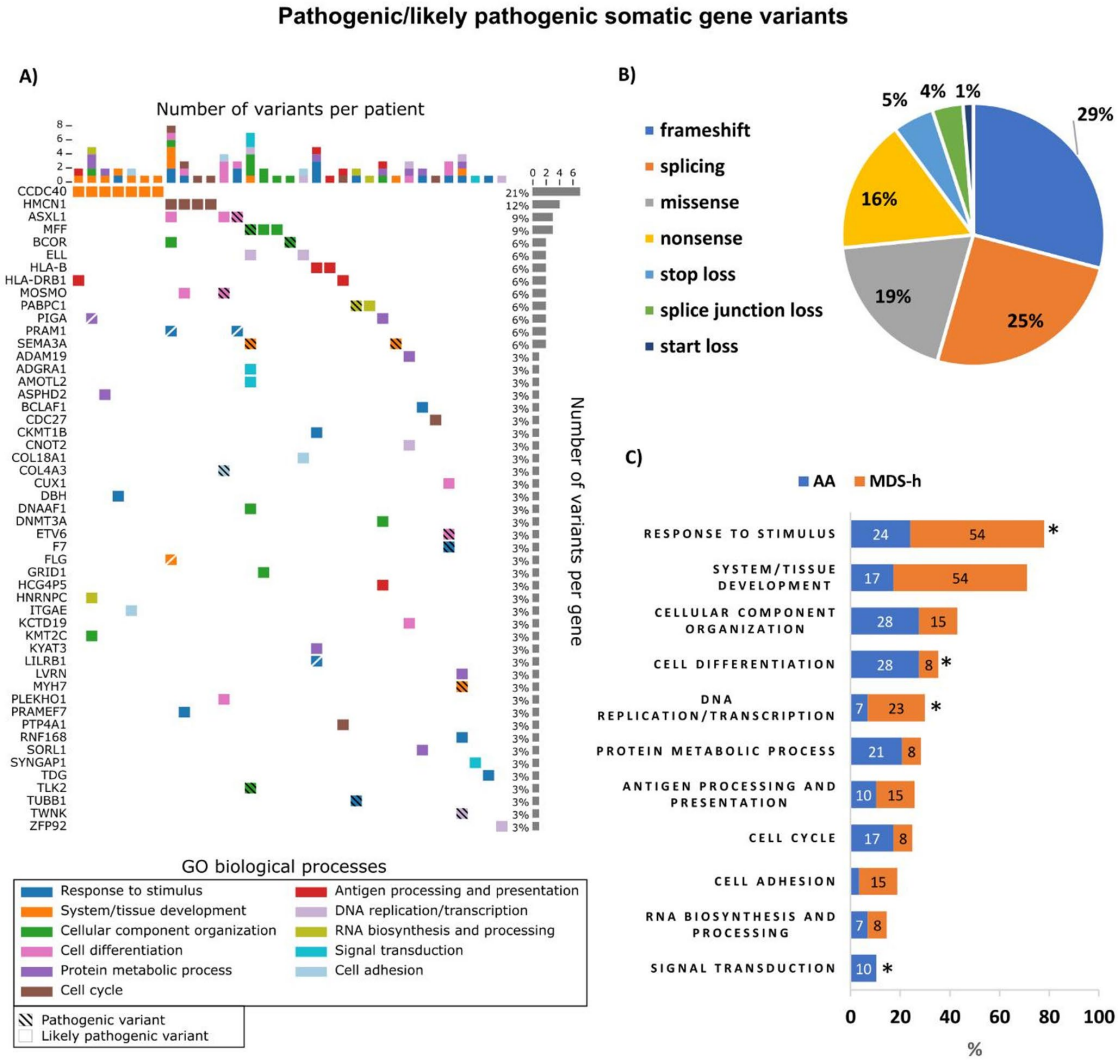
Pathogenic/likely pathogenic somatic gene variants in patients with AA and MDS-h. (A) Somatic mutation profiles of 33 patients at diagnosis. The figure illustrates the number of variants per patient (x-axis) and the number of variants per gene (y-axis). The colors of the individual squares represent the Gene Ontology (GO) biological process (BP) category terms. Hatched and unhatched boxes represent pathogenic and likely pathogenic variants, respectively. Split boxes indicate multiple variants in the gene. Only GO terms including 3 or more somatic variants are shown. (B) Distribution of somatic gene variants according to coding impact. (C) Frequency of somatic gene variants in AA vs. MDS-h patients according to the biological functionalities of the mutated genes. * *p* value < 0.05

A total of 89 nonsynonymous P/LP (15/74) somatic gene variants with a variant allele frequency (VAF) ≥ 0.10 were detected in 33 patients (79%) (Fig. 1A). The full list of P/LP somatic gene variants and their characteristics is available in Table S4. The majority of the variants were single nucleotide variants (SNVs) (42%), followed by deletions (30%), insertions (20%), and substitutions (8%). In terms of coding impact, the most common somatic variants were frameshift variants (29%), with the remainder being splicing (25%), missense (19%), and nonsense variants (16%) (Fig. 1B). On average, there were 2.2 somatic variants per AA patient (range 0–7) and 2.2 variants per MDS-h patient (range 0–9). The average VAFs of the somatic variants were 0.22 (median 0.14) and 0.23 (median 0.15) in the AA and MDS-h patients, respectively.

Eight myelodysplasia-associated somatic variants in the *BCOR/BCORL1*,* DNMT3A*,* ASXL1*,* CUX1*, and *ETV6* genes were found in 7 patients (5 AA and 2 MDS-h patients, 17%); neither AA patient had evidence of myelodysplasia at the time of analysis. The co-occurrence of two different myelodysplasia-associated somatic variants *(CUX1/ETV6* and *ASXL1/BCOR*) was observed in 2 patients. *PIGA* variants were detected only in 2 AA patients, one of whom carried 2 variants (NM_002641.4:c.403G > T(p.Ala135Ser) and NM_002641.4:c.380 C > A(p.Ser127Ter)) in this gene.

Twenty patients were also evaluated by targeted sequencing of a gene panel including 54 genes associated with myeloid malignancies. In 4 patients, targeted sequencing identified somatic variants in the *BCOR, BCORL1, DNMT3A*, and *ASXL1* genes at 0.05–0.73 VAF. All variants detected by targeted sequencing were also detected by WES, and VAF values (even below the threshold of 0.10 VAF for WES) were highly correlated (r = 0.97).

The functional annotation of somatically mutated genes clustered the somatic variants into 11 biological processes (BPs), including BMF-relevant processes such as antigen processing and presentation, and response to stimulus covering T cell-mediated immunity and DNA repair. Different BPs were asymmetrically distributed between AA and MDS-h patients (Fig. 1C). Acquired frameshift and nonsense variants in the *HLA-A, HLA-B*, and *HLA-DRB1* genes were detected in 5 patients (12%). Furthermore, 4 patients (9.5%) carried two concurrent variants in the *PRAM1* (*n* = 2), *LILRB1* (*n* = 1), and *BLACF1* (*n* = 1) genes involved in the adaptive immune response.

### Germline Mutation Landscape

**Fig. 2:**
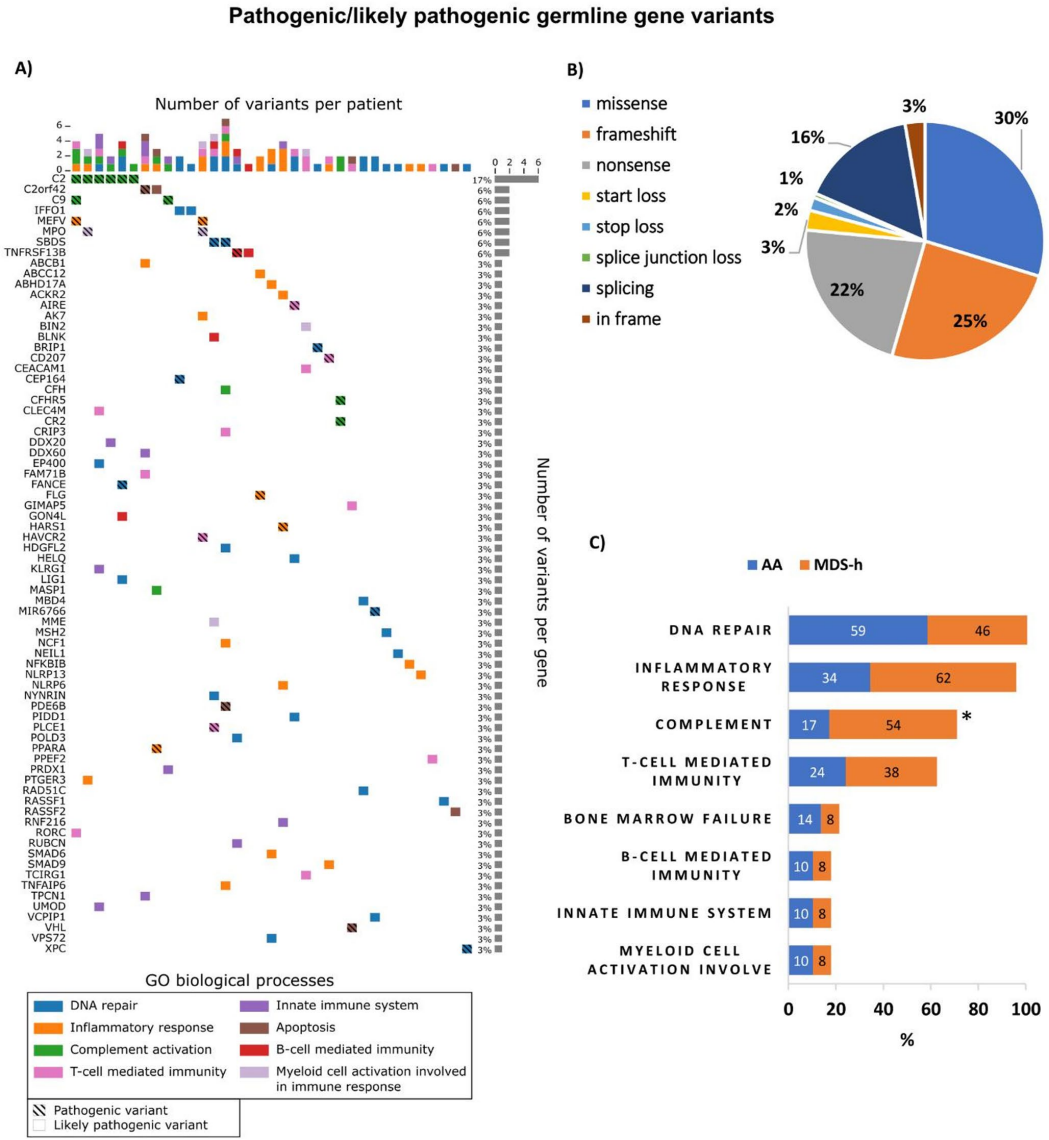
Pathogenic/likely pathogenic germline gene variants in patients with AA and MDS-h. (A) Germline mutation profiles of 42 patients at diagnosis. The figure illustrates the number of variants per patient (x-axis) and the number of variants per gene (y-axis). The colors of the individual squares represent the Gene Ontology (GO) biological process (BP) category terms. Hatched and unhatched boxes represent pathogenic and likely pathogenic variants, respectively. Split boxes indicate multiple variants in the gene. GO terms including only one germline variant are not shown. (B) Distribution of germline gene variants according to coding impact. (C) Frequency of germline gene variants in AA vs. MDS-h patients according to the biological functionalities of the mutated genes. Only GO terms with presumed functional consequences are shown. * *p* value < 0.05

At ≥ 0.35 VAF, a total of 352 nonsynonymous P/LP (97/255) germline variants were identified in all patients (Fig. 2A). A full list of P/LP germline gene variants and their characteristics is available in Table S5. Except for the *CR2* gene variant, all P/LP germline variants were heterozygous, and 61% (213/352) of the variants were in genes associated with autosomal dominant (AD) conditions (18 variants), autosomal recessive (AR) conditions (141 variants), AD/AR conditions (51 variants), or X-linked recessive conditions (3 variants). The majority of variants were SNVs (68%), and the remainder were insertions (10%) or deletions (22%). In terms of coding impact, the most frequent variants were missense variants (30%), followed by frameshift (25%), nonsense (22%), and splicing variants (16%) (Fig. 2B). The average number of germline variants per patient was 8.4, and MDS-h patients carried significantly more germline variants than did AA patients (9.6 vs. 7.8, *p* < 0.05). Germline variants in genes associated with inherited BMF syndromes (iBMFS), namely, *FANCE*,* BRIP1* (*FANCJ*), *SBDS*, and *RAD51C*, were found in 5 patients (12%). 

Concerning the biological functions of the affected genes, the greatest number of P/LP germline variants were located in genes involved in DNA repair (*n* = 23) and cell adhesion (*n* = 23), etc. (Fig. 2C) (Additional file 3: Fig. S2). A significant proportion of patients (45%) harbored germline variants in genes associated with inborn errors of immunity (IEI), such as *AIRE*,* CFH*,* CFHR5*,* CR2*,* C2*,* C9*,* MASP1*,* LIG1*,* TNFRSF13B*, and *BLNK*. Germline variants affecting complement genes were more prevalent in MDS-h patients than in AA patients (54% vs. 17%, *p* < 0.05). The *C2* variant (NM_000063.6(C2):c.841_849 + 19del) was the most frequent germline variant in our cohort (6/42, 14%) and its presence in BM and BS samples was validated by Sanger sequencing.

### Transcriptome Profiles of Peripheral Blood T Cells

**Fig. 3:**
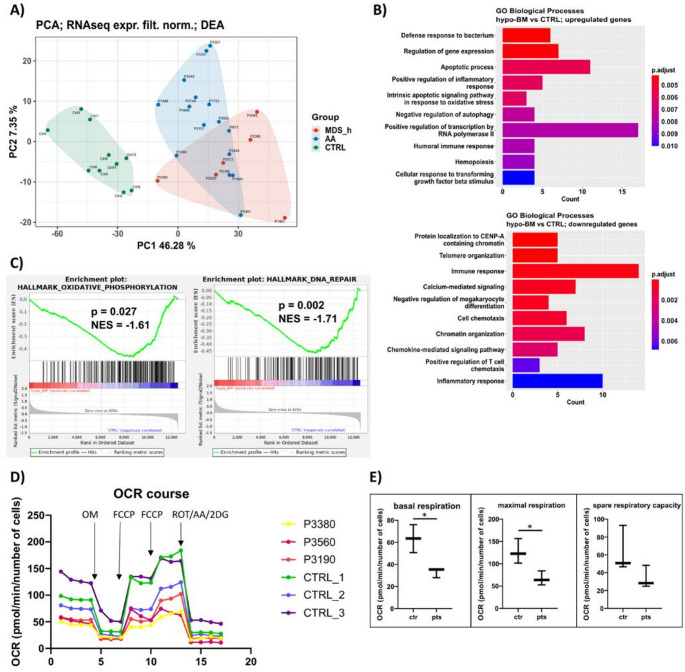
Transcriptomic and metabolic analysis of T cells from hypo-BM patients. (A) Principal component analysis (PCA) plot illustrating RNA-seq data from AA and MDS-h patients and healthy controls (CTRL). (B) The most significantly enriched Gene Ontology (GO) terms among the upregulated (upper plot) and downregulated (lower plot) genes in the T cells from hypo-BM patients compared with those from healthy controls (CTRL) (*p* < 0.05). The colors indicate the level of significance. (C) GSEA plots for Hallmark gene sets showing significant enrichment in controls versus hypo-BM patients. (D) Real-time measurements of the oxygen consumption rate (OCR) in hypo-BM patients and healthy controls. Each point in the lines represents the average measurement of three different wells. (E) Results from the quantitative analysis of the following cellular respiration parameters: basal respiration, maximal respiration, and spare respiratory capacity. *p < 0.05. AA, patients with aplastic anemia; MDS-h, patients with hypoplastic MDS; hypo-BM, AA plus MDS-h patients; ES, enrichment score; NES normalized enrichment score; OCR, oxygen consumption rate; OM, oligomycin; FCCP, carbonyl cyanide-p-trifluoromethoxyphenylhydrazone; ROT/AA/2DG, rotenone, antimycin A, 2-deoxyglucose mix

Peripheral blood T cells from 15 AA patients, 6 MDS-h patients, and 10 age-matched controls were subjected to transcriptome analysis using RNA-seq. We identified 12,595 transcripts, including 10,771 protein-coding genes (PCGs) and 1,181 long noncoding RNAs (lncRNAs), among all patients. Principal component analysis-based clustering of the RNA-seq data defined three major components for AA patients, MDS-h patients, and controls (Fig. 3A). As expected, the control samples formed a separate group, whereas the patient samples partially clustered together.

### Dysregulated Protein-coding Genes

Differential expression analysis identified 142 upregulated and 218 downregulated PCGs (|logFC|>0.5, FDR < 0.05) in hypo-BM patients compared with controls (Table S6). Gene Ontology analysis annotated the upregulated genes to 38 BP terms (*p* < 0.05). REVIGO integrated these terms into 31 groups, the top 10 of which included apoptotic process, positive regulation of inflammatory response, and intrinsic apoptotic signaling pathway in response to oxidative stress (Fig. 3B) (Table S7).

The downregulated genes were significantly enriched in BPs related to telomere organization, immune response, and chromatin organization, etc. (*p* < 0.05) (Fig. 3B) (Table S7).

Gene set enrichment analysis (GSEA) using the GO Biological Process (C5_BP) and Hallmark (H) gene sets revealed significant enrichment of the Oxidative Phosphorylation (C5_BP, H) and DNA Repair (H) gene sets in the control phenotype compared with the patient phenotype (|NES|>1.5, *p* < 0.05) (Fig. 3C). Suppressed oxidative phosphorylation (OXPHOS) was functionally confirmed by measuring mitochondrial respiration in T cells. The results revealed significantly lower basal *p* = 0.02) and maximal (*p* = 0.03) respiration in hypo-BM patients (*n* = 3) than in healthy controls (*n* = 3), indicating a reduction in OXPHOS (Fig. 3D, E).

### Long Noncoding RNA-target Gene Regulatory Network

**Fig. 4:**
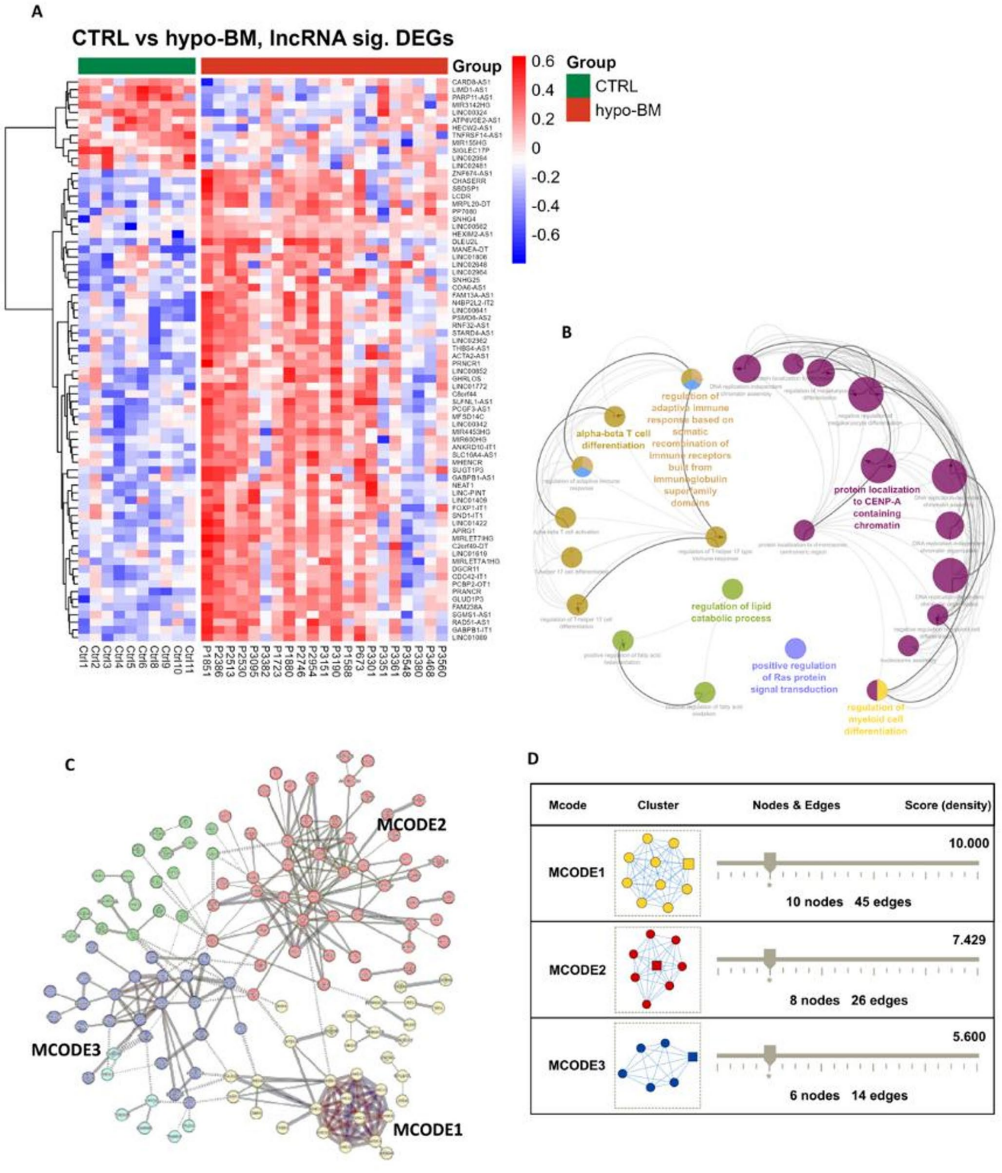
Dysregulated lncRNAs and their potential target genes in the T cells from hypo-BM-patients. (A) Heatmap of differentially expressed lncRNAs between hypo-BM patients and controls. The relative lncRNA expression changes are expressed by a color gradient intensity scale, as shown on the right. Blue indicates relatively low expression, while red indicates relatively high expression values. Each column represents a separate sample and each row a single lncRNA (only known lncRNAs are shown). (B) Functionally grouped networks of enriched Gene Ontology (GO) biological process terms generated for the target genes via ClueGO. The GO terms are represented as nodes, and the node size indicates the significance of the term enrichment. The node color indicates the class of the GO term. (C) Protein‒protein interaction (PPI) network of target genes constructed via STRING. The nodes indicate the proteins, and the edges indicate the interactions between two proteins. The colors indicate k-means (obsolete) clusters of proteins. The dotted lines indicate interactions between clusters. (D) Significant modules extracted from the PPI network by MCODE analysis in Cytoscape. CTRL, controls; hypo-BM, patients with hypoplastic bone marrow; P, patient

In T cells, a total of 194 differentially expressed lncRNAs (DElncRNAs) (|logFC|>0.5, FDR < 0.05) were identified by comparing hypo-BM patients to healthy controls. There were 173 upregulated and 21 downregulated lncRNAs in hypo-BM patients (Table S8). The heatmap of known DElncRNAs is shown in Fig. 4A.

To explore the potential biological functions of the DElncRNAs, GO term enrichment analysis was performed based on the annotations of differentially expressed mRNAs (DEmRNAs) that were significantly coexpressed with DElncRNAs (|R|≥ 0.6) [14]. The top 10 DEmRNAs with the strongest correlation for each DElncRNA were used for functional annotation (Table S9, S10) [15]. The nonredundant GO terms (FDR < 0.05) were visualized in functionally grouped networks using ClueGO (Fig. 4B). The analysis revealed that the BPs were primarily associated with the regulation of T helper 17 (Th17) type immune response, protein localization to chromosome - centromeric region, regulation of lipid catabolic process, regulation of myeloid cell differentiation, and positive regulation of Ras protein signal transduction.

The interaction relationships between target genes were outlined by the constructed protein-protein interaction (PPI) network (Fig. 4C). The three significant modules considered as key functional subnetworks were extracted from the PPI network by MCODE. These modules were related to chromatin structure (MCODE1), cytokine-cytokine receptor interaction (MCODE2), and RNA splicing (MCODE3) (Fig. 4D) (Table S11).

To search for cis-acting lncRNAs, neighboring DEmRNAs were identified by sliding 400 kb upstream and downstream of each DElncRNA [16, 17]. This screening resulted in 6 DElncRNA–DEmRNA pairs with both strong coregulation and genomic proximity (Table 1, Fig. S3).

**Table 1:** Cis-acting lncRNAs and their potential target genes in the T cells from hypo-BM patients

Cis-acting lncRNA	LncBook Gene ID	LncRNA regulation	Genomic location relative to target gene	Target gene	Gene regulation	Biological functionality	Correlation
ENSG00000290989	HSALNG0010926	Up	Downstream	WDR26	Up	Cell cycle progression	0.886
SLFNL1-AS1	HSALNG0002928	Up	Upstream	CTPS1	Up	T cell proliferation	0.870
ENSG00000285999	HSALNG0042313	Up	Downstream	SREK1	Up	RNA splicing	0.863
ENSG00000279168	HSALNG0060188	Up	Upstream	POLR2J3	Up	DNA-templated transcription	0.853
ENSG00000287932	HSALNG0059552	Up	Intra	CDK6	Up	Cell cycle progression	0.828
THBS4-AS1	HSALNG0042960	Up	Downstream	SERINC5	Up	Type I IFN production	0.788

### Gene Expression Signatures Associated with IST Response

**Fig. 5:**
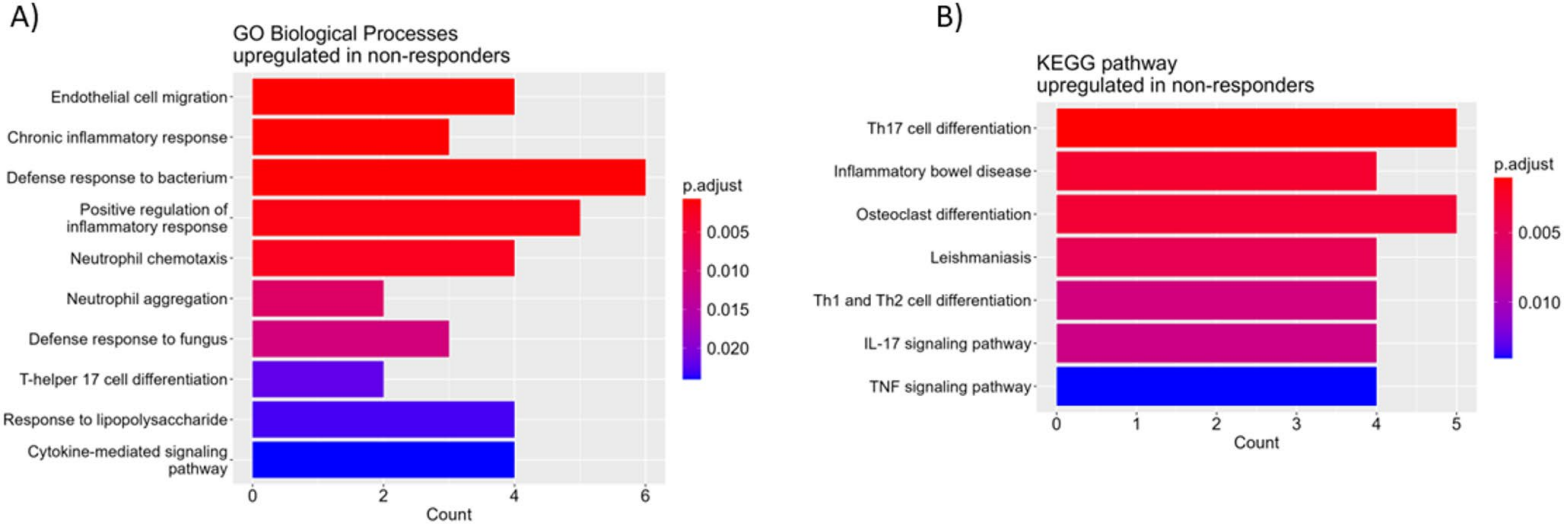
Gene Ontology enrichment analysis of dysregulated genes in the T cells from IST non-responders. The most significantly enriched GO biological processes and pathways among the upregulated genes in IST non-responders compared with responders (*p* < 0.05). The colors indicate the level of significance. IST, immunosuppressive therapy

To better understand IST resistance, we also compared T cell expression profiles between IST responders (RES) and non-responders (NR). Of the 21 patients analyzed by RNA sequencing, 12 received IST, of whom 7 responded after 6 months (ORR = 58%). The comparative analysis of the T cell profiles revealed 143 upregulated and 117 downregulated genes (|logFC|>0.5, FDR < 0.05) in NR compared to RES (Table S12). In NR, functional annotation of the upregulated genes identified enriched BP terms related mostly to inflammatory response, neutrophil activity, and Th17 cell differentiation (*p* < 0.05) (Fig. 5A). The downregulated genes showed enrichment in BPs associated with aerobic respiration, natural killer cell mediated cytotoxicity, granzyme-mediated programmed cell death signaling pathway, and immune response (*p* < 0.05) (Table S13).

According to the KEGG database, the upregulated genes were significantly overrepresented in signaling pathways relevant to T cell activation, such as Th17, Th1 and Th2 cell differentiation pathways, and IL-17 signaling pathway, in NR (*p* < 0.05) (Fig. 5B). In contrast, OXPHOS pathway was downregulated in NR (*p* < 0.05) (Table S13).

### Composition of Peripheral Blood Cell Populations

To study the imbalance in the composition of the PB cell populations, the frequencies of HSPCs, specific T cell subsets, and natural killer (NK) cells were measured in 14 hypo-BM patients and 6 healthy controls (Table S14). There was evident depletion of the pool of both immature (0.002% vs. 0.012%) and mature (0.017% vs. 0.079%) progenitors in AA patients compared with controls (*p* < 0.01*)*, and MSD-h patients exhibited depletion of immature progenitors only (0.005% vs. 0.012%, *p* < 0.01*)*. Concerning the T cell subsets, AA patients had a lower frequency of γδ T cells than controls (3.6% vs. 7.6%, *p* < 0.05), and MDS-h patients presented an increased proportion of activated CD4^+^ T cells (6.4% vs. 4.0%, *p* < 0.05), including the HLA-DR^+^ naive subset (8.2% vs. 4.8%, *p* < 0.05). All patients had a significantly lower frequency of NK cells than the controls (7.3% vs. 15.1%, *p <* 0.05). Significant correlations between AA severity and cell population frequency were detected in immature and mature progenitors, Tregs, HLA-DR^+^ naive CD4^+^ T cells, and NK cells (*R* ≤ −0.65, *p* < 0.05). Additionally, the frequency of effector memory CD8^+^ T cells was associated with IST response (*R* = −0.65, *p* < 0.05), with higher frequencies detected in NR. Heatmap displaying correlation coefficients between various cell populations and key clinical/hematological parameters in AA is shown in Figure S4.

## Discussion

Whole-exome sequencing is a powerful tool for identifying new gene variants associated with diseases of unclear etiology. To gain more insight into the genomic landscape of aBMFS, we used comparative WES in AA and MDS-h patients to identify acquired and inherited gene variants in all coding regions. Somatic gene variants were found in 79% of AA and 77% of MDS-h patients, most of which were private, nonrecurrent variants. Compared to studies using NGS-based gene panels, our WES-based study did not reveal significant differences in the average number of somatic variants per patient or the average VAF between AA and MDS-h patients [18]. Myelodysplasia-associated somatic variants were found in similar proportions of AA and MDS-h patients and were associated with severe BM hypoplasia in all cases. This finding demonstrates the limitations of MDS-associated somatic mutations in the differential diagnosis of AA and MDS-h in patients with unsuccessful or unclear morphologic assessment due to the very low cellularity of BM aspirates.

Epidemiologic studies have reported an association of specific *HLA* class I alleles with an increased risk of aAA [19]. The *HLA* alleles are hypothesized to be the target antigens of autoreactive T cells, and 6pLOH leading to the loss of specific alleles is the mechanism by which HSPCs may escape T cell-mediated damage. In our cohort, we found 6pLOH in only 1 (4%) of the 24 patients tested by SNP array. Studies using targeted deep sequencing have further uncovered inactivating somatic mutations of *HLA* alleles as another possible origin of clonal escape [20]. Our WES data provide further evidence for this mechanism, as *HLA* mutations were detected in 12% of the patients; however, our patients also acquired alterations in the *HLA* class II gene (*HLA-DRB1*), indicating both HLA class I- and HLA class II-restricted mechanisms. In a previous study employing high-throughput genotyping of *HLA* loci in AA, the *HLA* class II *DQB1* locus was reported as the most frequently mutated locus and allelic loss events were predominantly observed in the *HLA* class II *DRB1* and *DQB1* loci [21]. Furthermore, deleterious somatic variants were detected in genes implicated in T cell signaling, such as *LILRB1*,* BLACF1*, and *PRAM1* [22]. In particular, LILRB1 interacts with MHC class I molecules and suppresses the autoreactive immune response. Defects in LILRB1 function have been associated with several autoimmune diseases [23]. Disruption of genes by deleterious mutations within the T cell activation pathway may impair the immune synapse required for proper T cell-mediated response and lead to overactivation of the immune system.

Using the WES method, P/LP germline gene variants were found in all patients in this study. Most of the variants were found in genes not associated with hematological or immunological diseases or with cellular pathways relevant to bone marrow failure, and were inherited in a recessive manner. Therefore, these variants are unlikely to have clinical relevance in the context of AA and MDS-h. In contrast, and consistent with the immunopathogenesis of aBMFS, nearly half of the patients had germline variants in genes that shape the immune response, including many genes associated with IEI (e.g., *AIRE*,* TNFRSF13B*,* BLNK*,* C2*,* C9*,* CR2*,* CFH* and *MASP1*). Notably, variants in genes that regulate immunological self-tolerance (*AIRE*,* TNFRSF13B*,* ITK*,* GIMAP5*,* HAVCR2*,* RORC*, and *TCIRG1*) may contribute to enhanced autoimmune responses.

Acquired or inherited complement deficiencies strongly favor the development of autoimmunity. We detected germline variants in several complement genes (*C2*,* C9*,* CFH*,* CFHR5*,* CR2*, and *MASP1*) and these variants were more prevalent in MDS-h than in AA. The heterozygous *C2* deletion variant was the most frequent germline variant in our cohort (6/42, 14%). Heterozygous *C2* variations in combination with variants in other complement genes (*C4* and *C8*) have been reported to increase the risk of autoimmune diseases such as systemic lupus erythematosus [24]. In our cohort, we found 2 patients with two concurrent germline complement variants each (*C2/C9* and *CR2/CFHR5*). The patient with *CR2*/*CFHR5* variants experienced recurrent life-threatening infections with the development of acute disseminated encephalomyelitis and did not respond to IST. Our data support the recent findings that germline gene variants predisposing to immunodeficiency are overrepresented in patients with acquired BMF and may be implicated in the pathogenesis of these immune cytopenias [25].

Many of the identified germline variants were located in genes involved in inflammation, which is driven by an imbalance in inflammatory cytokines in immune-mediated BMF. The inflammatory response category included mutated genes previously linked to autoinflammatory diseases (*MEFV* and *ABCB1*) or genes encoding proteins with anti-inflammatory capacity (*PPARA*,* NLRP6*,* NFKBIB*,* AK-7*, and *SMAD6)* whose disruption may enhance autoinflammation.

In aBMFS, proper DNA repair is crucial for the survival and self-renewal of HSPCs that are exposed to damage by autoreactive T cells. Moreover, the residual HSPCs of AA patients exhibit increased susceptibility to genomic instability, as evidenced by the enrichment of altered isoform usage in DNA repair genes [26]. In this context, we identified multiple germline variants in DNA repair genes, such as the *FANCE*,* FANCJ*,* SBDS*,* RAD51C*, *XPC*,* IFFO1*,* LIG1*,* MBD4*, and *POLD3* genes, that may contribute to DNA damage in HSPCs in AA and MDS-h.

Inherited BMFS comprises a group of heterogeneous disorders with genetic etiologies. Here, heterozygous germline variants in iBMFS-associated genes were found in 12% of patients who had no family history of iBMFS. Similar frequencies of aAA patients carrying iBMFS-associated variants have been reported by others, who have further demonstrated that these carriers have a poor response to IST [27, 28]. There is growing evidence that certain germline heterozygous recessive variants in dominant genes may contribute to the development of BMF [29–31]. Heterozygous mutations in Fanconi anemia genes predispose AA and MDS patients to BMF and leukemogenesis [32], and heterozygosity for the 258 + 2 T >C *SBDS* mutation appears to be another genetic factor accelerating telomere shortening in aAA patients [33]. These findings highlight the importance of considering germline genetic factors in the evaluation of AA and MDS-h patients since iBMFS-variant carriers may be at greater risk of disease progression.

To study the molecular mechanisms underlying the abnormal T cell response in immune-mediated BMF, we performed transcriptome profiling of T cells from hypo-BM patients. Functional annotation of the dysregulated PCGs revealed that patient T cells presented expression signatures associated with increased inflammation, apoptosis, and hypoxia response and decreased oxidative stress-induced apoptosis relative to those from healthy controls. In addition, GSEA specifically revealed significant suppression of OXPHOS in patient T cells. We therefore focused on investigating OXPHOS-related changes. Metabolic assay confirmed a significant reduction in both mitochondrial basal and maximal respiration in the T cells of hypo-BM patients compared with those of controls, indicating their altered bioenergetic state. Our results support the theory that effector T cells tend to utilize aerobic glycolysis to meet their high energy demands during their activation and expansion phases [34]. These findings highlight the potential therapeutic implications of targeting metabolic pathways in T cells. Agents such as 2-deoxyglucose, an inhibitor of glycolysis, can prevent the differentiation of T cells into Th17 cells, a subset of proinflammatory T cells that has been implicated in autoimmune and inflammatory diseases [35].

Despite the importance of lncRNAs in the regulation of hematopoiesis and immunity, lncRNAs have received little attention in research on acquired BMF. To date, there are limited data on lncRNAs in aAA and no data specifically for the MDS-h subtype. We analyzed the lncRNA profiles of T cells from hypo-BM patients and searched for DElncRNAs and their putative target genes. The identified DElncRNAs with known biological functions included lncRNAs involved in BMF-relevant processes such as T cell activation (*NEAT1* and *PCBP2-OT1*), the inflammatory response (*THBS4-AS1* and *FOXP1-IT1*), and clonal hematopoiesis (*SNHG4*,* GABPB1-AS1*,* LINC-PINT*, and *NEAT1*), indicating a BMF-specific lncRNA network.

Since lncRNAs regulate gene expression by modulating mRNAs, the biological functions of DElncRNAs were predicted based on the functional annotation of coregulated DEmRNAs. Consistent with the major role of lncRNAs in gene expression regulation through chromatin remodeling, the histone gene cluster emerged as the most significant module in the PPI network. The histone genes were predicted to be regulated by *HEXIM2-AS1*,* N4BP2L2-IT2*,* FAM13A-AS1*, and *THBS4-AS1*, suggesting their involvement in chromatin structure modulation. The analysis further revealed that lncRNAs may play a vital role in T cell dysregulation, mainly by regulating the differentiation of Th17 cells. Our data suggest that specific lncRNAs (*ATP6V0E2-AS1* and *HECW2-AS1*) may regulate Th17 cell differentiation by targeting the *IL23A* gene. IL-23 is an inflammatory cytokine that drives Th17 cell expansion by activating the JAK-STAT3 pathway, particularly STAT3, which promotes the expression of Th17-specific genes [36]. Further, the expansion of activated T cells may be enhanced by markedly upregulated expression of *CTPS1* likely regulated by cis-acting *SLFNL1-AS1*. All patients also showed strong upregulation of *NEAT1*, which may enhance the activation of STAT3 signaling pathway, resulting in Th17 cell differentiation [37]. Due to this role, elevated *NEAT1* levels may serve as a potential biomarker for immune activation and inflammation in BMF disorders.

Many lncRNAs function via cis-acting mechanisms and regulate neighboring target genes. Our screening for cis-regulated target genes identified several strong candidates whose biological functions are closely related to abnormal T cell features observed in acquired BMF, such as T cell expansion (*CTPS1*), IFN expression (*SERINC5*), and cell cycle progression (*CDK6* and *WDR26*), and thus serve as targets for our future research.

We further aimed to identify the dysregulated cellular pathways that may drive resistance to IST and contribute to persistent BMF in non-responding patients. The transcriptomic profiling of T cells from RES and NR revealed a significant upregulation of genes associated with Th17 cell differentiation and IL-17 signaling in NR. In parallel, the upregulated pathways detected in NR that are linked to Th1 and Th2 differentiation further reflect the imbalance in T cell subsets that contributes to ongoing immune activation in NR. The downregulated genes in NR point to impaired innate immune responses, including natural killer cell cytotoxicity and granzyme-mediated apoptosis, which may compromise the resolution of inflammation. In NR, the suppression of OXPHOS-related processes likely reflects enhanced T cell activation and expansion driven by increased reliance on glycolysis. These findings highlight the central role of Th17-driven immune dysregulation in the pathogenesis of refractory AA and indicate that the Th17/IL-17 axis represents the most important therapeutic target in patients unresponsive to conventional IST.

In immune-mediated BMF, the bone marrow is typically empty due to HSPC destruction by autoreactive T cells. Using flow cytometry, we confirmed the depletion of the pool of immature progenitors in all hypo-BM patients. In patients with aAA, previous studies have shown that the frequency of effector CD8^+^ T cells is increased due to oligoclonal expansion, while the frequency and function of regulatory T cells (Tregs) are decreased [38, 39]. In our patients, the frequencies of all the CD8^+^ cell subsets were comparable to those of controls, and only AA patients had a lower frequency of Tregs, but this difference was not statistically significant (*p* = 0.08). However, when NSAA patients were excluded from the analysis, we found a highly significant difference between SAA/VSAA patients and controls (*p* < 0.01), indicating a correlation between the Treg fraction and disease severity. This was confirmed by correlating Treg frequencies with AA subtypes. Additionally, we observed significant correlation between effector memory CD8⁺ T cell frequency and IST response, with higher frequencies in NR. Similar findings have been reported, highlighting the expansion of effector memory CD8⁺ T cells in refractory AA and their association with worse outcome and treatment response [39].

Compared with controls, all AA patients also presented a very low frequency of γδ T cells. Reduced γδ T cells, together with increased levels of cell subsets producing interleukin (IL)−17 A, are likely to mediate autoreactive T cell activation in SAA [40]. Our study also provides further evidence for the involvement of impaired innate immunity in the pathophysiology of immune-mediated BMF, as the number of NK cells was markedly decreased in all hypo-BM patients and correlated with AA severity. Reduced NK cell number and function with recovery of cytotoxicity after successful IST have been previously observed in SAA patients, suggesting their protective role against disease onset [41].

We acknowledge that the relatively small sample size of our cohorts may limit the statistical power of our findings. This limitation prevents us from evaluating disease subtype-specific mutation and gene expression patterns, which would enhance the clinical and biological relevance of our results.

In conclusion, our WES-based study revealed that the somatic mutation profiles of AA and MDS-h patients display considerable diversity, with a low number of recurrently mutated genes. The study highlights overrepresentation of germline immune-related gene variants in patients with acquired BMF, providing a basis for understanding genetic predispositions. These pathogenic variants may increase susceptibility to immune-mediated BMF or affect the “immune fitness” of patients, and thus represent potential risk factors for progressive cytopenia and/or IST failure.

Transcriptomic and metabolic analysis of patient T cells uncovered significant suppression of OXPHOS, indicating changes in bioenergetics of T cells. Interaction network data further revealed the involvement of lncRNAs in the aberrant T cell response, primarily via the regulation of Th17 cell differentiation. To our knowledge, this is the first report providing lncRNA patterns of T cells specifically from MDS-h and adult AA patients.

## Supplementary Information

Below is the link to the electronic supplementary material.

Supplementary Material 1(XLSX72.3 KB)

Supplementary Material 2(XLSX 123 KB )

Supplementary Material 3(DOCX.76 MB)

Supplementary Material 2

Supplementary Material 3

## Data Availability

The datasets generated and/or analysed during the current study are available in the National Center for Biotechnology Information (NCBI) Sequence Read Archive (SRA) database under accession number PRJNA1216066 (WES data) (PRJNA1216066 - SRA - NCBI) and PRJNA1206975 (RNA-seq data) (PRJNA1206975 - SRA - NCBI).
