# Supplementary material for: Overrepresentation of Germline Immune-Related Gene Variants in Patients with Acquired Bone Marrow Failure
Source: J Clin Immunol. 2025 Nov 5;45(1):150. doi: 10.1007/s10875-025-01951-6 (PMC12586405; doi:10.1007/s10875-025-01951-6)
Supplement: Supplementary file 3 — Supplementary Material 3(DOCX.76 MB) [file 10875_2025_1951_MOESM3_ESM.docx]

1. **SUPPLEMENTARY** **METHODS**

**DNA sequencing**

Variant allele frequency (VAF) thresholds of ≥0.10 and ≥0.35 were used for somatic and germline variants, respectively. The clinical significance of the sequence variants was classified according to the American College of Medical Genetics and Genomics (ACMG). Only pathogenic (P) and likely pathogenic (LP) variants were analyzed further. All identified P/LP variants were manually reviewed.

All detected germline variants were manually reviewed and classified according to the American College of Medical Genetics and Genomics/Association for Molecular Pathology (ACMG/AMP) guidelines for the interpretation of sequence variants [1]. For each variant, the relevant ACMG/AMP criteria were assessed based on the available evidence, including population allele frequencies, computational predictions, functional studies, and previously reported pathogenicity (ClinVar submissions). A detailed summary of criteria for each variant is listed in Supplementary Table S4 and S5.

Gene variants were classified as germline if detected in both bone marrow and matched buccal swab samples. The sequence variant NM_000063.6(C2):c.841_849+19del was verified by Sanger sequencing as described previously [2]. The sequencing data were deposited in the National Center for Biotechnology Information (NCBI) Sequence Read Archive (SRA) database (<https://www.ncbi.nlm.nih.gov/sra>) under accession number PRJNA1216066.

**RNA sequencing**

The RNA samples were ribodepleted prior to library preparation using the RiboCop rRNA Depletion Kit (Lexogen, Wien, Austria). Functional annotation of differentially expressed (DE) transcripts was performed using the DAVID Knowledgebase (v2024q2) and KEGG database (v112.0) [3,4]. The Gene Ontology (GO) terms were summarized using the REVIGO Web server (v1.8.1) and visualized by ClueGO (v2.5.10) [5]. Gene set enrichment analysis (GSEA) was performed using online application (<https://cloud.genepattern.org/gp/pages/index.jsf>). Curated gene set libraries C2, C5 and Hallmarks from MSigDB (<https://www.gsea-msigdb.org/gsea/msigdb>) were used to find significantly enriched gene sets. A protein-protein interaction (PPI) network was constructed via the STRING database (v12.0) [6]. The MCODE plug-in (v2.0.3) for Cytoscape (v3.10.2) was used to extract significant subnetworks and to identify hub genes. The heatmap and PCA plot were exported from R software. The complete raw data were deposited in the NCBI SRA database under accession number PRJNA1206975 ([Homo sapiens (ID 1206975) - BioProject - NCBI](https://www.ncbi.nlm.nih.gov/bioproject/PRJNA1206975)).

**Seahorse Assay**

The experiment was performed according to a protocol described by *Trsova* et. al. [7]. Briefly, cells were seeded at a density of 200,000 cells per well in Agilent XFp PDL miniplates in XF RPMI Base Medium pH 7.4 supplemented with 1 mM pyruvate, 10 mM glucose and 2 mM L‐glutamine for the Mito Stress Test (all Agilent Technologies). The final concentrations of the injected drugs were 1 μM oligomycin, 2 μM FCCP, 0.5 μM rotenone + antimycin A and 50 mM 2‐deoxyglucose mixture (all Agilent Technologies).

**Flow cytometry**

Peripheral blood mononuclear cells (PBMC) from 14 hypo-BM patients and 6 healthy controls were thawed in RPMI 1640 (Sigma‒Aldrich, St. Louis, MO, USA) supplemented with 2% human serum albumin (Kedrion**,** Barga, Italy), 0.2% sodium bicarbonate (Euroclone, Pero, Italy), and 200 µL/L Benzonase^®^ Nuclease (Merck, San Jose, CA, USA) to prevent cell clumping. The cells were incubated overnight in RPMI 1640 supplemented with 10% fetal bovine serum (Thermo Fisher Scientific, Waltham, MA, USA), 0.2% sodium bicarbonate (Euroclone), 1 mg/L folic acid (Sigma‒Aldrich), 0.4 mM HEPES (Lonza, Basel, Switzerland), 50 µM 2-mercaptoethanol (Sigma‒Aldrich), and 2 mM L-glutamine–penicillin–streptomycin solution (Merck) at 37°C in a 5% CO_2_ atmosphere.

The cells were counted, and 1–5×10^6^ cells per sample were stained. A total of 1×10^6^ healthy donor PBMCs or CompBeads (BD Biosciences, Milpitas, CA, USA) were used as compensation controls. Mononuclear cells from AML leukapheresis were used as controls for CD34 compensation. To control for viability, healthy PBMCs were heat-killed (70 °C, 5 min), and a mixture of stained and unstained killed cells was evaluated. Patient samples, control samples and single-stained compensation controls were processed in the same manner. Briefly, the cells were washed with 2 mL of PBS (Lonza) and stained with BD Horizon™ Fixable Viability Stain 575V (1 µL stain/1 mL sample in PBS; BD Biosciences], 0.1% NaN_3_ [Sigma‒Aldrich]_,_ 200 mg/L human IgG, and 1 mM EDTA [Duchefa Biochemie, BH Haarlem, Netherlands]) and stained with an antibody cocktail (Table S3) prepared with Brilliant Stain Buffer (BD Biosciences). After staining (30 min, at room temperature), the cells were washed with staining buffer and fixed with Intracellular Fixation Buffer (Thermo Fisher Scientific) for 20 min. Finally, the cells were washed with CellWASH buffer (BD Biosciences) and resuspended in 400 µL of CellWASH buffer for measurement.

The samples were measured on a BD FACSymphony A5 cytometer equipped with 5 lasers and 31 detectors, and the data were analyzed using FlowJo 10.8.1 software (BD Biosciences). The cell populations were defined as shown in Figure S1.

**2. SUPPLEMENTARY TABLES**

**Table S1. Clinical and laboratory characteristics of AA and MDS-h patients.**

| **Characteristics** | **AA** | **MDS-h** | ***p* value** |
| --- | --- | --- | --- |
| No. of patients | 37 | 18 |  |
| Age (years) (median, range) | 48 (19-74) | 37 (22-81) | *0.87* |
| Gender (male/female) | 19/18 | 8/10 | *0.39* |
| Neutrophils (x10^9^/L) (median, range) | 1.04 (0,01-4.69) | 0.66 (0.07-4.05) | *0.35* |
| Platelets (x10^9^/L) (median, range) | 19 (3-211) | 36 (3-167) | *0.88* |
| Reticulocytes (%) (median, range) | 0.98 (0.17-4.18) | 1.36 (0.6-3.58) | *0.47* |
| Hemoglobin (g/L) (median, range) | 90 (47-167) | 93 (64-149) | *0.71* |
| BM blasts (%) (median, range) | 0 (0-3.0) | 0.4 (0-3.8) | *0.08* |
| PNH clone presence (n) (%) | 20/33 (60%) | 4/13 (31%) | ***0.04*** |
| Elevated LDH (n) (%) | 10/37 (27%) | 2/18 (11%) | *0.09* |
| Abnormal conventional cytogenetics (n) (%) | 2/31 (6.5%) | 1/16 (6 %) | *0.48* |
| Abnormal SNP-A karyotyping (n) (%) | 3/16 (19%) | 1/8 (13%) | *0.30* |
| MDS-associated somatic mutations (n) (%) | 8/20 (40%) | 4/12 (33%) | *0.50* |

AA, aplastic anemia; MDS-h, hypoplastic myelodysplastic neoplasms; BM, bone marrow; PNH, paroxysmal nocturnal hemoglobinuria; LDH, lactate dehydrogenase; SNP-A, single-nucleotide polymorphism array; MDS, myelodysplastic neoplasms.

Bold *p* values indicate a statistically significant difference (*p* <0.05) between AA and MDS-h patients.

**Table S2. IST response evaluation in AA patients: relationship with baseline clinical and laboratory characteristics.**

| **Characteristics** | **Responders** | **Non-responders** | ***p* value** |
| --- | --- | --- | --- |
| No. of patients | 18 | 6 |  |
| Age (years) (median, range) | 27 (19-68) | 67 (30-74) | ***0.01*** |
| Gender (male/female) | 11/7 | 0/7 | ***0.004*** |
| AA severity (n) (%) |  |  | *0.09* |
| NSAA | 8 (44) | 1 (17) |  |
| SAA | 9 (50) | 4 (66) |  |
| VSAA | 1 (6) | 1 (17) |  |
| Overall survival (months) (median, range) | 97 (8-196) | 24 (6-39) | ***0.02*** |
| Neutrophils (x10^9^/L) (median, range) | 2.44 (0.36-4.69) | 0.43 (0.19-2.44) | ***0.006*** |
| Platelets (x10^9^/L) (median, range) | 65 (4-211) | 22 (3-95) | ***0.05*** |
| Hemoglobin (g/L) (median, range) | 106 (52-167) | 77 (64-98) | ***0.01*** |
| BM blasts (%) (median, range) | 0.4 (0-3.0) | 0.0 (0-0.5) | *0.08* |
| PNH clone presence (n) (%) | 8 (44%) | 4 (66%) | *0.25* |
| PNH clone size (n) (%) |  |  | *0.20* |
| 0-1% | 7 (39%) | 3 (50%) |  |
| 1-10% | 1 (6%) | 1 (17%) |  |
| Abnormal conventional cytogenetics (n) (%) | 0 (0%) | 1 (17%) | *0.08* |
| MDS-associated somatic mutations (n) (%) | 5 (28%) | 3 (50%) | *0.36* |

IST, immunosuppressive treatment; AA, aplastic anemia; NSAA, non-severe AA; SAA, severe AA; VSAA, very severe AA; BM, bone marrow; PNH, paroxysmal nocturnal hemoglobinuria; MDS, myelodysplastic neoplasms.

Bold *p* values indicate a statistically significant difference (*p* <0.05) between responders and non-responders.

A total of 24 patients were treated with IST (see Table S2). Eighteen of the 24 evaluable patients responded after 6 months of treatment (ORR=75%), including 12 complete responses (50%). Overall, responders were significantly younger and demonstrated superior overall survival, as well as improved hematological parameters (specifically higher hemoglobin levels, neutrophil and platelet counts), compared to non-responders. In contrast, no significant association was observed between IST response and the presence or size of a PNH clone or MDS-associated mutations. Furthermore, the distribution of AA subtypes did not differ significantly between responders and non-responders.

**Table S3: List of antibodies used for flow cytometry panel.**

| **Target** | **Conjugate** | **Clone** | **Isotype** | **Manufacturer** | **Cat. Number** | **µL/100 µL staining volume** |
| --- | --- | --- | --- | --- | --- | --- |
| CD4 | BUV395 | SK3 | Mouse BALB/c IgG1, κ | BD Biosciences | 563550 | 5 |
| CD8 | BUV496 | RPA-T8 | Mouse IgG1, κ | BD Biosciences | 612943 | 5 |
| TCRγδ | BUV615 | 11F2 | Mouse BALB/c IgG1 | BD Biosciences | 751308 | 5 |
| CD45 | BUV805 | HI30 | Mouse IgG1, κ | BD Biosciences | 612891 | 3 |
| CD38 | BV421 | HIT2 | Mouse IgG1, κ | BD Biosciences | 562444 | 1 |
| CD127 | BV605 | HIL-7R-M21 | Mouse IgG1, κ | BD Biosciences | 562662 | 5 |
| CD27 | BV650 | L128 | Mouse BALB/c IgG1 | BD Biosciences | 563228 | 5 |
| CD28 | BV786 | L293 | Mouse BALB/c IgG1, κ | BD Biosciences | 742530 | 5 |
| CD45RA | BB515 | HI100 | Mouse IgG2b, κ | BD Biosciences | 564552 | 5 |
| CD34 | PE | 581 | Mouse IgG1, κ | BD Biosciences | 555822 | 10 |
| CD25 | PE-CF594 | M-A251 | Mouse BALB/c IgG1, κ | BD Biosciences | 562403 | 5 |
| CD56 | PE-Cy7 | B159 | Mouse IgG1, κ | BD Biosciences | 557747 | 1 |
| CD16 | PE-Cy7 | 3G8 | Mouse BALB/c x DBA/2 | BD Biosciences | 557744 | 0.5 |
| CCR7 | APC | 2-L1-A | Mouse BALB/c IgG1, κ | BD Biosciences | 566762 | 2 |
| HLA-DR | R718 | L203.rMAb | Mouse BALB/c IgG1, κ | BD Biosciences | 752501 | 1 |
| CD3 | APC-H7 | SK7 | Mouse BALB/c IgG1, κ | BD Biosciences | 560176 | 5 |

**Tables S4 and S5** are provided as separate Excel files due to the large size of the datasets.

**Table S6. Differentially expressed protein-coding genes in the T cells from healthy controls compared with those from hypo-BM patients (|logFC|>0.5, FDR<0.05).** hypo-BM, hypocellular bone marrow; logFC, log fold change; FDR, false discovery rate.

**Table S7. Gene Ontology (GO) term enrichment analysis of up- and downregulated protein-coding genes in the T cells from hypo-BM patients (p<0.05).** GO terms assigned to biological processes are sorted by decreasing GO enrichment test p value. hypo-BM, hypocellular bone marrow.

**Table S8. Differentially expressed long noncoding RNAs in the T cells from healthy controls compared with those from hypo-BM patients (|logFC|>0.5, FDR<0.05).** hypo-BM, hypocellular bone marrow; logFC, log fold change; FDR, false discovery rate.

**Tables S9** is provided as separate Excel files due to the large size of the dataset.

**Table S10. Gene Ontology (GO) term enrichment analysis of mRNAs significantly coexpressed with lncRNAs in the T cells from hypo-BM patients (p<0.05).** GO terms assigned to biological processes are sorted by decreasing GO enrichment test p value.

**Table S11. Molecular complex detection (MCODE) clusters for significant modules from protein-protein interaction network of the target genes in the T cells from hypo-BM patients.**

**Table S12. Differentially expressed genes in the T cells from IST non-responders (NR) compared with those from responders (RES) (|logFC|>0.5, FDR<0.05).** IST, immunosuppressive therapy; logFC, log fold change; FDR, false discovery rate.

**Table S13. Gene Ontology (GO) term enrichment analysis of up- and downregulated genes in the T cells from IST non-responders (p<0.05).** TOP 10 GO terms assigned to biological processes and signaling pathways are sorted by decreasing GO enrichment test p value. IST, immunosuppressive therapy.

**Table S14: Frequencies of specific cell subpopulations in peripheral blood mononuclear cells isolated from AA and MDS-h patients and healthy blood donors.** Values are expressed as mean ± standard deviation. The AA and MDS-h groups were compared with the control group. Statistical significance is indicated as: *p <0.05; ** p <0.01. AA, aplastic anemia; MDS-h, hypoplastic myelodysplastic neoplasms; CTRL, controls; NK, natural killer.

**3. SUPPLEMENTARY FIGURES**

**Fig. S1: Schematic of the cell population definition strategy.
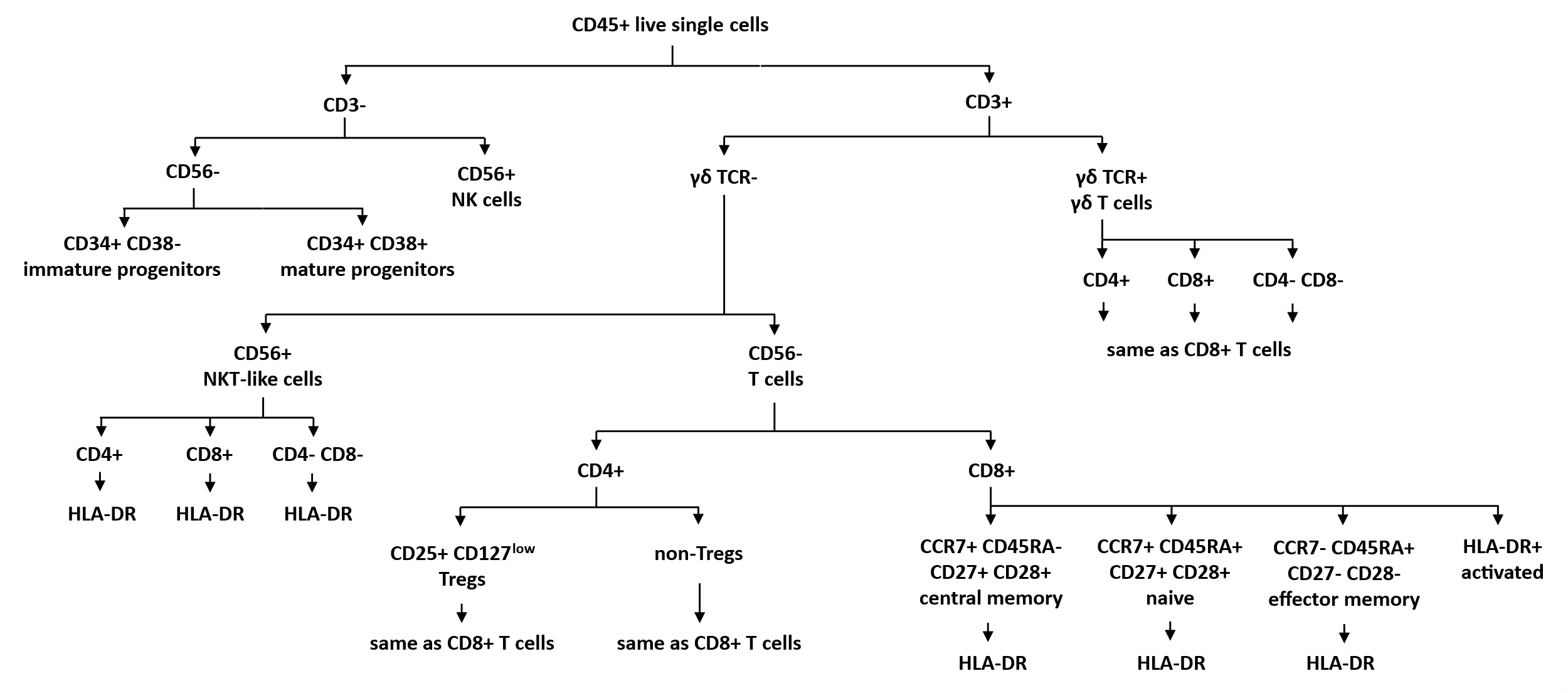
**

 **Fig. S2: GO biological processes with the highest number of genes disrupted by germline variants.**


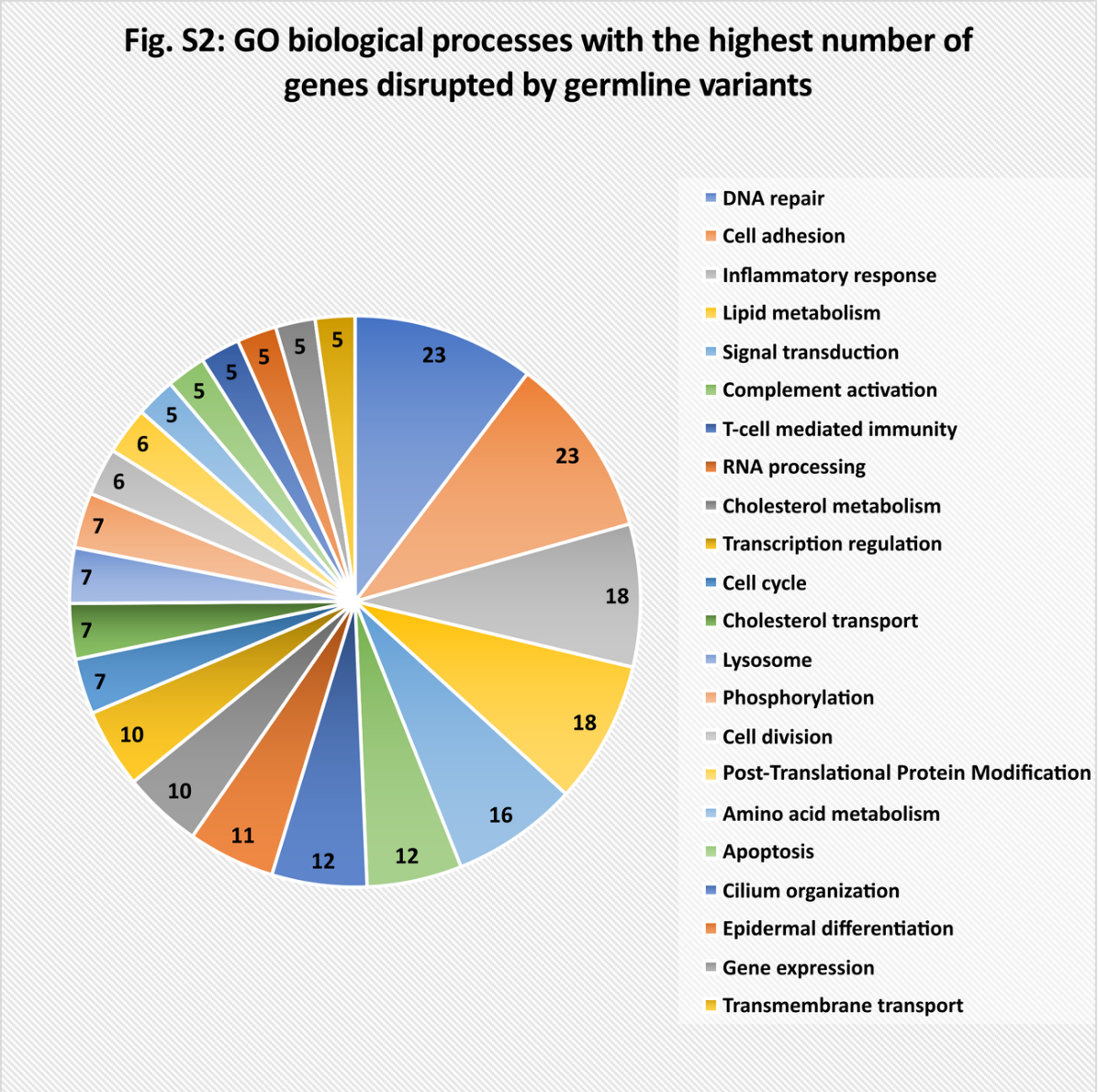


**Fig. S3: Correlation curves depicting significant correlations of cis-acting lncRNA and target gene expression levels.** Green and red dots indicate controls and patients, respectively. CTRL, controls; hypo-BM, patients with hypoplastic bone marrow; R, Pearson correlation coefficient; CPM, counts per million; p, *p* value.


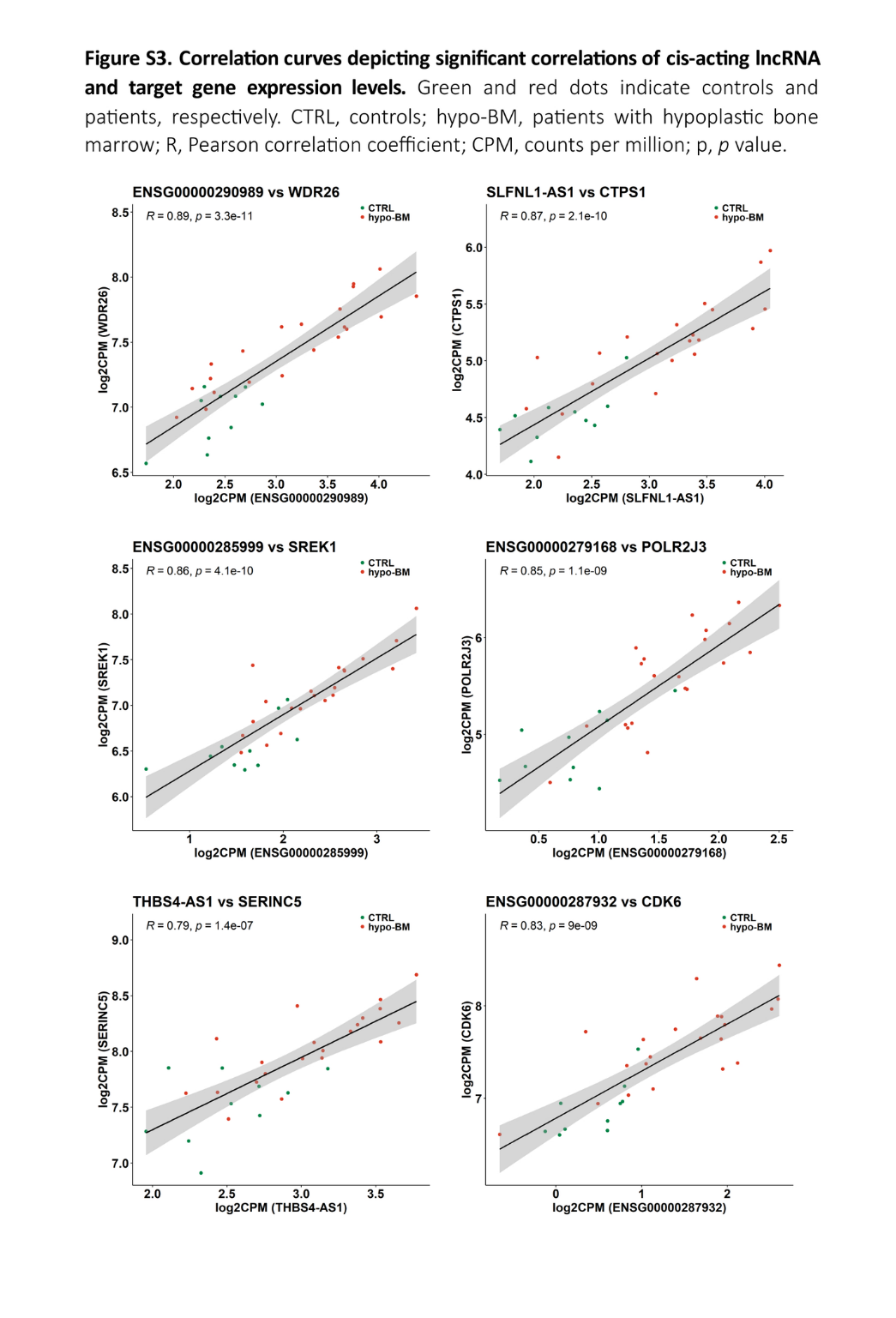


**Fig. S4. Heatmap showing correlation coefficients between various cell populations and key clinical/hematological parameters in AA.** The color scale represents the strength and direction of the correlation, with red indicating positive and blue indicating negative correlations.


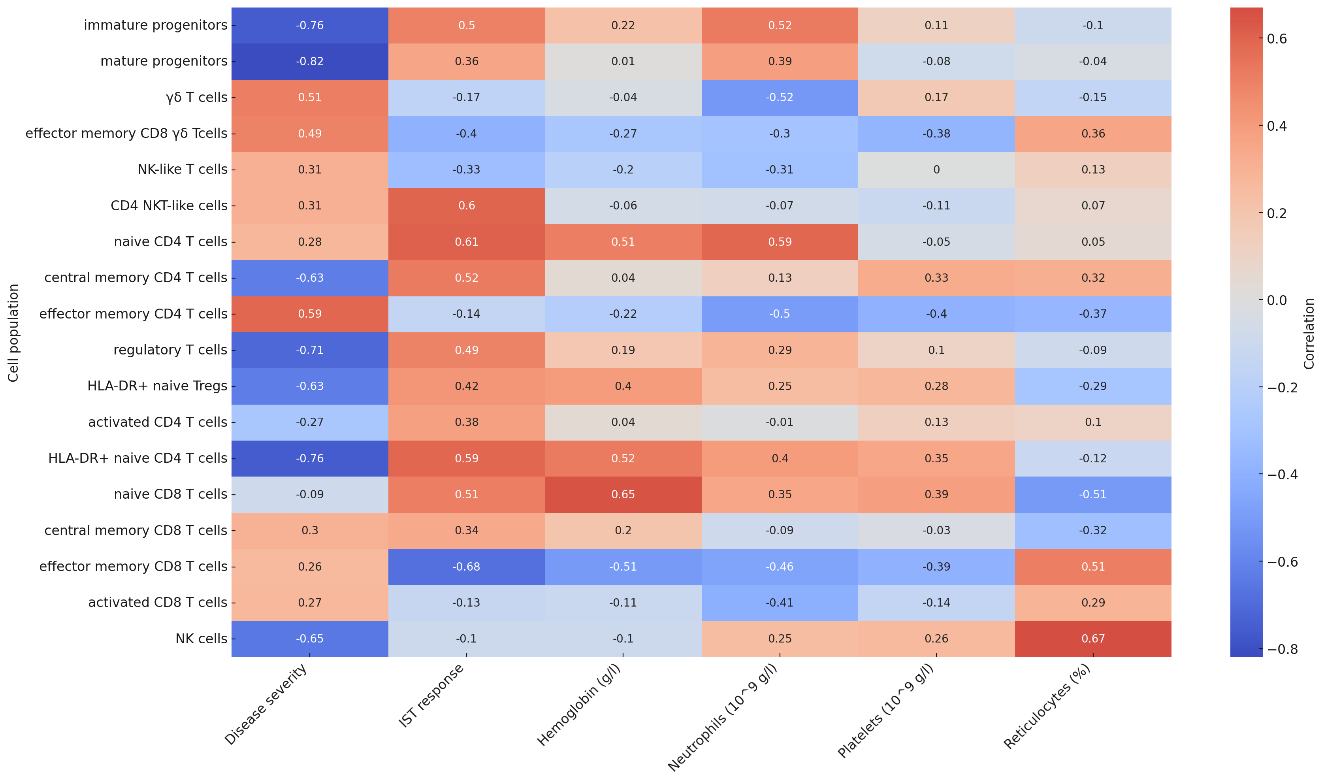


4. **SUPPLEMENTARY** **REFERENCES**

1. Comm ALQA, Richards S, Aziz N, Bale S, Bick D, Das S, et al. Standards and guidelines for the interpretation of sequence variants: a joint consensus recommendation of the American College of Medical Genetics and Genomics and the Association for Molecular Pathology. GENETICS IN MEDICINE. 2015;17(5):405–24.

2. Kaisrlikova M, Vesela J, Kundrat D, Votavova H, Merkerova MD, Krejcik Z, et al. *RUNX1* mutations contribute to the progression of MDS due to disruption of antitumor cellular defense: a study on patients with lower-risk MDS. Leukemia. 2022;36(7):1898–906.

3. Jiao XL, Sherman BT, Huang DW, Stephens R, Baseler MW, Lane HC, et al. DAVID-WS: a stateful web service to facilitate gene/protein list analysis. BIOINFORMATICS. 2012;28(13):1805–6.

4. Kanehisa M, Sato Y, Kawashima M, Furumichi M, Tanabe M. KEGG as a reference resource for gene and protein annotation. Nucleic Acids Res. 2016;44(D1):D457–62.

5. Supek F, Bosnjak M, Skunca N, Smuc T. REVIGO Summarizes and Visualizes Long Lists of Gene Ontology Terms. PLoS One. 2011;6(7).

6. Szklarczyk D, Kirsch R, Koutrouli M, Nastou K, Mehryary F, Hachilif R, et al. The STRING database in 2023: protein-protein association networks and functional enrichment analyses for any sequenced genome of interest. Nucleic Acids Res. 2023;51(D1):D638–46.

7. Trsova I, Hrustincova A, Krejcik Z, Kundrat D, Holoubek A, Staflova K, et al. Expression of circular RNAs in myelodysplastic neoplasms and their association with mutations in the splicing factor gene *SF3B1*. Mol Oncol. 2023;17(12):2565–83.
